# Expression Analyses of Embryogenesis-Associated Genes during Somatic Embryogenesis of *Adiantum capillus-veneris* L. *In vitro*: New Insights into the Evolution of Reproductive Organs in Land Plants

**DOI:** 10.3389/fpls.2017.00658

**Published:** 2017-04-27

**Authors:** Xia Li, Jing-Dan Han, Yu-Han Fang, Shu-Nong Bai, Guang-Yuan Rao

**Affiliations:** ^1^RDFZ XiShan SchoolBeijing, China; ^2^School of Life Sciences, Peking UniversityBeijing, China

**Keywords:** *Adiantum capillus-veneris*, somatic embryogenesis, embryogenesis-associated genes, gene expression, phylogenetic analysis

## Abstract

An efficient *in vitro* regeneration system *via* somatic embryogenesis (SE) was developed for a fern species *Adiantum capillus-veneris*. Adventitious shoots, green globular bodies (GGBs) and calli were obtained with the maximal induction rate on the Murashige and Skoog (MS) medium of low concentrations of 6-benzyladenine (BA) (0–1.0 mg/L), 2.0 mg/L BA without 2,4-dichlorophenoxyacetic acid (2,4-D), 0.5 mg/L 2,4-D and 0.5–1.0 mg/L 6-BA, respectively. Cyto-morphological and histological changes in the shoot development *via* calli and GGBs were examined. For a better understanding of these developmental events, expression patterns of six genes, *AcLBD16, AcAGL, AcBBM, AcWUS, AcRKD*, and *AcLEC1*, were characterized during SE. *AcBBM* and *AcLEC1* were ubiquitously expressed in direct SE (adventitious shoots and GGBs) the maximal expression of *AcBBM* in mature GGBs, and the high expression of *AcLEC1* in GGB initiation and adventitious shoots. During the indirect SE, *AcLBD16, AcLEC1, AcRKD*, and *AcWUS* were highly expressed in mature calli. Additionally, phylogenetic analyses showed that *AcWUS, AcBBM, AcLBD, AcAGL, AcRKD*, and their homologs of other green plants formed monophyletic clades, respectively. Some of these gene families, however, diversified rapidly with the occurrence of embryophytes, suggesting that embryogenesis-associated genes could experience a rapid evolution with the colonization of plants to terrestrial environments. Expression and phylogenetic analyses of those embryogenesis-associated genes by the aid of *in vitro* regeneration system of *A. capillus-veneris* provide new insights into the evolution of reproductive organs in land plants.

## Introduction

The occurrence of embryos was presumably one of the most significant innovations during plant evolution, which is crucial for plant reproduction (Kenrick and Crane, 1997; Becker and Marin, 2009; Pires and Dolan, 2012; Radoeva and Weijers, 2014). Obviously, embryogenesis is a defining feature of land plants, and establishes the basic body plan of sporophytes. In addition to zygote-derived embryogenesis, other modes of embryogenesis have been described, such as somatic embryogenesis (SE). SE is the biological process through which a whole individual is regenerated from somatic tissues via their dedifferentiation and redifferentiation (Fehér, 2015; Loyola-Vargas and Ochoa-Alejo, 2016). Not only is SE one of the most powerful tools in plant biotechnology, but it also becomes an efficient approach to study the mechanisms of the embryo development (Radoeva and Weijers, 2014; Fehér, 2015; Loyola-Vargas and Ochoa-Alejo, 2016).

SE can be divided into two types: one is the direct SE where the embryos are formed from the organized tissue without an intervening callus stage; another is the indirect SE where the embryo formation experiences a callus phase (Radoeva and Weijers, 2014). Genetic studies in the past decades have identified and cloned the genes and loci required for initiation and progression of zygotic embryogenesis (ZE) in Arabidopsis (Tzafrir et al., 2004; Radoeva and Weijers, 2014; Mikuła et al., 2015), but not in non-seed plants. According to previous studies, the similarity in the regulatory mechanisms that underlie SE and ZE has recently become evident at the molecular level (Tzafrir et al., 2004; Radoeva and Weijers, 2014; Loyola-Vargas and Ochoa-Alejo, 2016). Thus, the SE system can be used to investigate the progression and morphogenetic events during embryogenesis of early land plants, and also to examine the ZE regulatory mechanisms by analyzing the expression pattern of embryogenesis-associated genes during their SE. These data will provide insights into the evolution of reproductive organs in land plants.

Although the tissue culture of ferns has been studied for decades (Beck and Caponetti, 1983; Fernández et al., 1997; Bertrand et al., 1999; Fernández and Revilla, 2003; Liao and Wu, 2011; Mallón et al., 2011), only a few species, such as the tree fern *Cyathea delgadii*, was described concerning SE (Mikuła et al., 2015). As ferns are the closest living relatives of spermatophytes (Pryer et al., 2001), this group of plants has been useful subjects for evolutionary, morphological and developmental studies (Johnson and Renzaglia, 2009; Li et al., 2013; Vasco et al., 2013). In terms of SE, ferns have been apparently under-investigated compared to spermatophytes, and the molecular mechanism underlying the control of SE is poorly understood.

Recently, efforts have been made to describe SE at the molecular level in seed plants, and several groups of genes associated with this process have been revealed. PINs, Aux/IAAs, AUXIN RESPONSE FACTORS (ARFs) as well as LATERAL ORGAN BOUNDARIES DOMAIN (LBD) family have been shown to be involved in auxin generating somatic embryos (Jenik and Barton, 2005; Leyser, 2005). In addition, a series of transcription regulator genes are strongly implicated in this process. Among members of the APETALA2/ethylene-responsive factor (AP2/ERF) family, BABY BOOM (BBM) is a key transcription regulator that has been detected to involve the induction of SE (Boutilier et al., 2002). AGAMOUS like-15 (AGL15), a MADS-domain transcription factor, is involved in meristem development and also functions as a transcriptional activator during somatic embryo formation (Harding et al., 2003). WUSCHEL (WUS) and WUS-related homeobox domain (WOX), which are homeodomain containing transcription factors, regulate stem cell fate during embryo formation and have also been detected during SE (Zuo et al., 2002; Iwase et al., 2011). The RKD (RWP-RK domain-containing) proteins, such as RKD1, RKD2, and RKD4, are another class of transcription factors involved in early embryogenesis and female gametogenesis (Koszegi et al., 2011).

As a leptosporangiate fern, *Adiantum capillus-veneris* has become the subject of many cytological, developmental, physiological, and phylogenetic studies (Pryer et al., 2001; Wada, 2007; Xie et al., 2008; Li et al., 2013). Although there are several literatures on the direct SE of *A. capillus-veneris*, few of them have presented sufficient histological evidence to describe this process (Salomé et al., 1985; Amaki and Higuchi, 1991; Somer et al., 2010). In addition, the genes required to regulate callus and GGB induction and development are not well defined in *A. capillus-veneris*. It is also believed that expression analyses of the genes regulating SE can provide insights into this developmental process (Chugh and Khurana, 2002; Stasolla et al., 2004).

In the present work, we established a regeneration system *via* SE for *A. capillus-veneris* and three types of regeneration structure, i.e., shoots, GGBs and calli, were obtained. The main developmental events leading to the generation of shoots from calli and GGBs of *A. capillus-veneris* were examined by histological analyses. For a better understanding of the genes and regulatory mechanisms behind the fern SE, six homologous genes of Arabidopsis *LBD16, WUS, LEC1, RKD4, AGL15, BBM* were identified and cloned from *A. capillus-veneris*. Meanwhile, phylogenetic analyses of those genes of the main lineages of land plants including bryophytes, monilophytes, gymnosperms and angiosperms were conducted for their molecular evolution. In addition, the expression patterns of those genes were characterized during the crucial steps of the SE *via* GGBs and calli. All of the above analyses will shed insights into the evolution of reproductive organs in land plants.

## Materials and methods

### Plant induction system

For the induction system, explants of circinate leaflets from the sporophytes of *A. capillus-veneris* cultivated in the greenhouse of Peking University (Beijing, China) were cultured on full strength Murashige and Skoog medium (MS) or ½ MS medium supplemented with different concentrations of 2,4-dichlorophenoxyacetic acid (2,4-D) and 6-benzyladenine (BA) as follows: 0/0, 0.5/0, 1.0/0, 1.5/0, 2.0/0; 0/0.5, 0.5/0.5, 1.0/0.5, 1.5/0.5, 2.0/0.5; 0/1.0, 0.5/1.0, 1.0/1.0, 1.5/1.0, 2.0/1.0; 0/1.5, 0.5/1.5, 1.0/1.5, 1.5/1.5, 2.0/1.5; 0/2.0, 0.5/2.0, 1.0/2.0, 1.0/2.0, 1.5/2.0, and 2.0/2.0 mg/L. All cultures were maintained at 25 ± 1°C under a 16/8 h (light/dark) cycle and then transferred into induction medium ~30 days later for further observation and proliferation.

### Somatic embryogenesis induction and statistical analysis

The shoots and green global bodies (GGBs) were generated from direct SE, whereas calli were produced by indirect SE according to the aforementioned SE's definition (Table S1). The GGBs continuously increased in size in the selected medium. When the size of GGBs was 8–10 mm in diameter, they were cut into 3–4 pieces (~3 mm in diameter) and then transplanted into the same medium subculture. In parallel, primary calli from the best induction medium (>80%) were cut into the same small size (~3 mm in diameter) and then transplanted into their original initiation medium.

GGBs were transferred to shoot induction medium containing 0.5 mg/L BA under aseptic conditions. Shoots were observed at ~20 days. When most of the shoots had several leaves, they were transplanted into the PGR-free MS medium. For shoot induction from calli, MS and ½ MS medium with 0, 0.5, 1.0, 1.5, or 2.0 mg/L BA were chosen as the regeneration media. Calli were excised to ~3 mm in diameter and subcultured in the regeneration medium, which was renewed every 20 days. After 3 months of subculture, the total number of shoots per callus was calculated.

To induce roots, multiple shoot clusters were cut into small pieces (~5 mm in diameter) and then transferred to ½ MS medium supplemented with 0.5 mg/L naphthaleneacetic acid (NAA). After incubation for a total of 40 days, the well-developed plantlets were gently washed to remove agar and transferred from the culture flask to plastic pots containing vermiculite and nutrition soil [1:1 (v/v)].

The induction of shoots, GGBs and calli were repeated two times, with each replicate comprising 6–15 explants in a single Petri plate. The differentiation of shoots from calli involved 10 calli and three experimental replicates were conducted. All data were analyzed using one-way ANOVA followed by Duncan's multiple range test with significance level of *P* < 0.05 (IBM_ SPSS ver. 16).

### Microscopic preparation

For histological characterization, samples were fixed in formalin-alcohol-acetic acid (50% ethanol: formaldehyde: acetic acid, 91:5:4) for >24 h. The samples were dehydrated in an ethanol series and then an alcohol-acetone series (ethanol: acetone, 2:1, 1:1, 1:2, 0:1, and 0:1, changed every 30-min). For semithin sections, the tissues were then subjected to an acetone-Spurr's resin series (acetone: resin, 2:1, 1:1, 1:2, 0:1, 0:1, and 0:1) changed every 8 h. Finally, the samples were embedded in Spurr's resin. Sections (3 μm thick) were cut using a microtome (Leitz, 1512, Germany) and stained with 2% basic fuchsin. Images were observed and captured with a light microscope (Zeiss Axioskop 2 Plus, Germany) coupled with Axioplan software. For scanning electron microscopy (SEM), the samples were critical-point dried in CO_2_ (HCP-2; Hitachi, Tokyo, Japan) for 6 h. The dried samples were mounted, sputter-coated with gold palladium (Hitachi E-1010), and viewed under a Hitachi S-4800 SEM at 10.0 kV.

### Gene cloning and sequencing

Arabidopsis LBD16 (AT2G42430), AGL15 (AT5G13790), BBM (AT5G17430), WUSCHEL (AT2g17950), and RKD4 (AT5G53040) protein sequences were used as queries to find the homologs in the RNA-Seq database of *A. capillus-veneris* (Li et al., unpublished data). All the homologs, designated AcLBD16, AcAGL, AcBBM, AcWUS, and AcRKD were verified by sequencing coding DNA sequences using appropriate primers (Table S2). All these genes and their sequence were submitted to GenBank under accession number KP238200–KP238204.

### Quantitative RT-PCR (qRT-PCR) analysis

For qRT-PCR analysis, nine samples were used to identify the genes' expression patterns. Explant leaflets and eight different tissues at adventitious shoot, GGB and callus developmental stages were chosen: the initiation stage (14-day), mature stage (40-day), callus-embryo transition stage and GGB-/callus-derived shoots. Total RNA was isolated with Plant RNA Extraction Reagent (Invitrogen, USA) and purified with an RNeasy Mini kit according to the manufacturer's instructions (Qiagen, Germany). RNA was then converted to cDNA by reverse transcription with a FastQuant RT Kit (Tiangen, China). qRT-PCR was performed on an Applied Biosystems 7500 Real-Time PCR System (ABI) with the reaction mixture containing cDNA templates, primers (Table S3) and SYBR® Premix Ex Tax Mix (Takara, Japan). Transcript levels were normalized against the *A. capillus-veneris* actin gene (*AcACTIN*) transcript levels using appropriate primers (Table S3). Relative expression was calculated *via* the delta-delta threshold method (2-^ΔΔC^T) (Livak and Schmittgen, 2001). Results were expressed as means ± SE (standard error) of two biological repeats.

### Phylogenetic analysis

To gain insight into the evolutionary relationships of AcLBD16, AcAGL, AcBBM, AcWUS, and AcRKD with their counterparts in other plants, we performed the exhaustive phylogenetic analyses of LBD gene superfamily, and AP2, MADS-box, WOX, and RKD gene family. The homologous amino acid sequences of *A. thaliana, Oryza sativa, Amborella trichopoda, Picea abies, A. capillus-veneris, Selaginella moellendorffii, Physcomitrella patens, Marchantia polymorpha*, and algae were retrieved from databases Phytozome (http://phytozome.jgi.doe.gov/pz/portal.html), ConGenIE (http://congenie.org), and the *Klebsormidium flaccidum* genome project (http://www.plantmorphogenesis.bio.titech.ac.jp/~algae_genome_project/klebsormidium/index.html) (Table S4). The sequences were aligned using the online version of MAFFT (http://mafft.cbrc.jp/alignment/software/) (Katoh and Standley, 2013). The final alignments were analyzed using Protest (Abascal et al., 2005) to choose the best-fit models at amino acid level for molecular evolution. Maximum likelihood (ML) phylogenetic analyses were performed with RaxML and statistically evaluated by the bootstrap method using 1000 replicates (Stamatakis et al., 2008). Trees were edited using FigTree v1.4.2. (http://tree.bio.ed.ac.uk/software/figtree/).

## Results

### Somatic embryogenesis induction system

**Figure 1:**
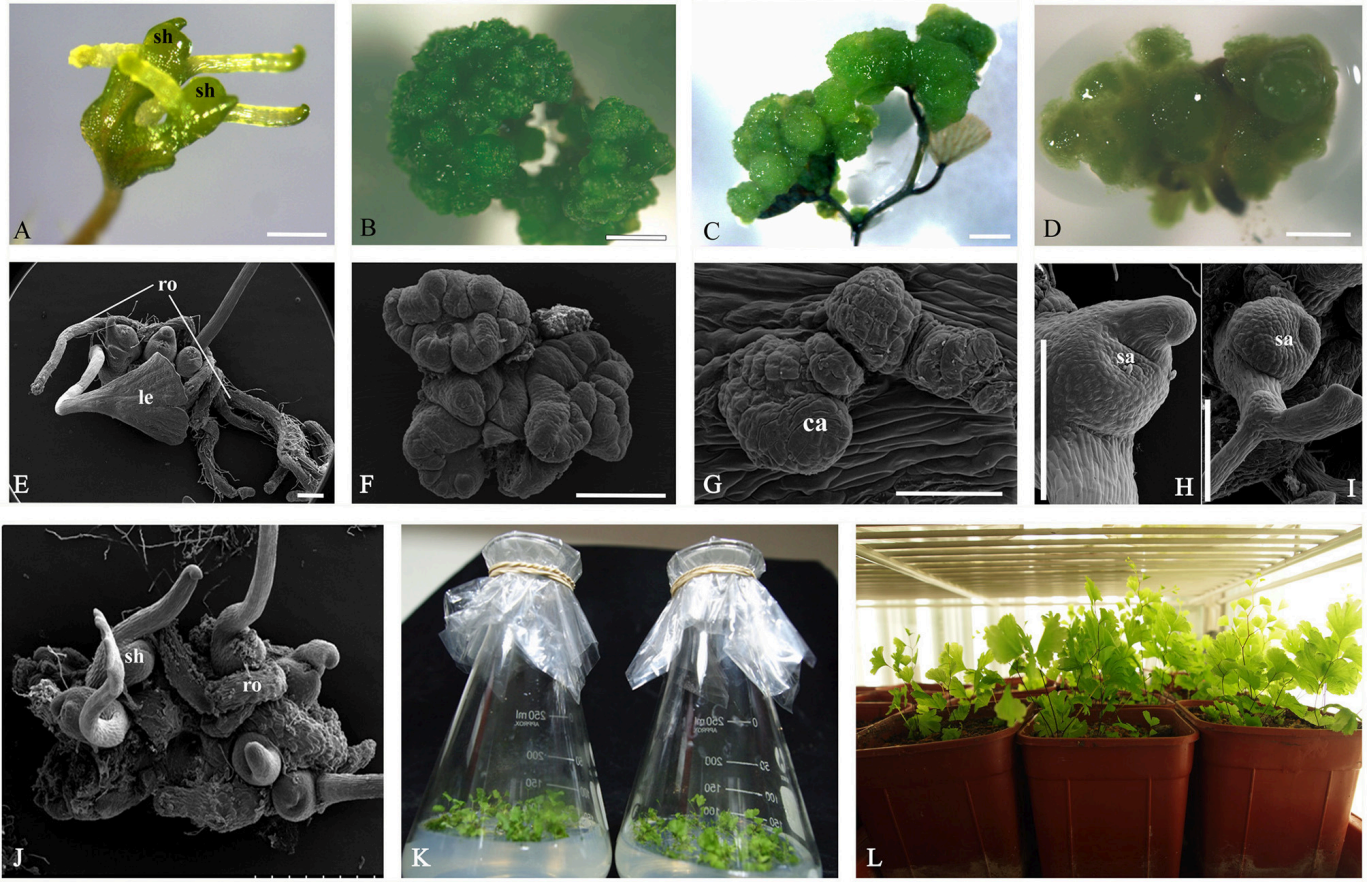
**Somatic embryogenesis induction system in *Adiantum capillus-veneris***. **(A,E)** Adventitious shoots formed on medium without PGR. **(B,E)** GGBs cultured on medium with 2.0 mg/L 6-BA. **(C,G)** Callus formed on medium with 0.5 mg/L 2, 4-D and 0.5 mg/L 6-BA. **(D)** Dispersed cells cultured on medium with 2.0 mg/L 2,4-D and 0.5 mg/L 6-BA. **(H)** Adventitious shoots derived from callus; **(I)** Shoots of sexual reproduction from archegonium. **(J)** Well-developed shoots. **(K)** Root formation of shoots on ½ MS medium with 0.5 mg/L NAA in flask; **(L)** Rooted plantlets transferred to pots for acclimatization. Ca, callus; sh, shoot; ro, root; le, leaf; sa, shoot apex. Bars = 500 μm.

Three induction systems of SE were produced by adventitious shoots, GGBs and calli when explants were cultured on the basal medium supplemented with different concentrations of 2,4-D and BA (Figure 1, Table S1). After ~2 weeks' culture, differentiated shoots were first observed on PGR-free media (Figures 1A,E). When the concentration of BA was increased to 1.5 mg/L and 2.0 mg/L, few shoots but GGBs were produced (Figures 1B,F). Under low concentrations of 2,4-D, a primary callus was induced after 2 weeks of culture (Figures 1C,G), while in higher concentrations of 2,4-D, e.g., from 1.5 to 2.0 mg/L, fewer calli were induced and they had a soft texture, dispersing cells on the medium (Figure 1D). When GGBs or calli were moved to the medium free of 2,4-D and with a lower concentration of BA, shoots readily formed within 5 months of incubation (Figure 1I). Well-developed shoots were transferred onto the rooting medium comprising ½ MS basal medium supplemented with 0.5 mg/L NAA to promote root formation (Figure 1J). Subsequently, more than 10 roots were produced after approximately 4 weeks of culture (Figure 1K). By this stage, plantlets had developed from the explants. When transplanted into soil, ~90% of the plantlets survived and produced spores 2 months later (Figure 1L).

### Cyto-morphological evidence for direct somatic embryogenesis

**Figure 2:**
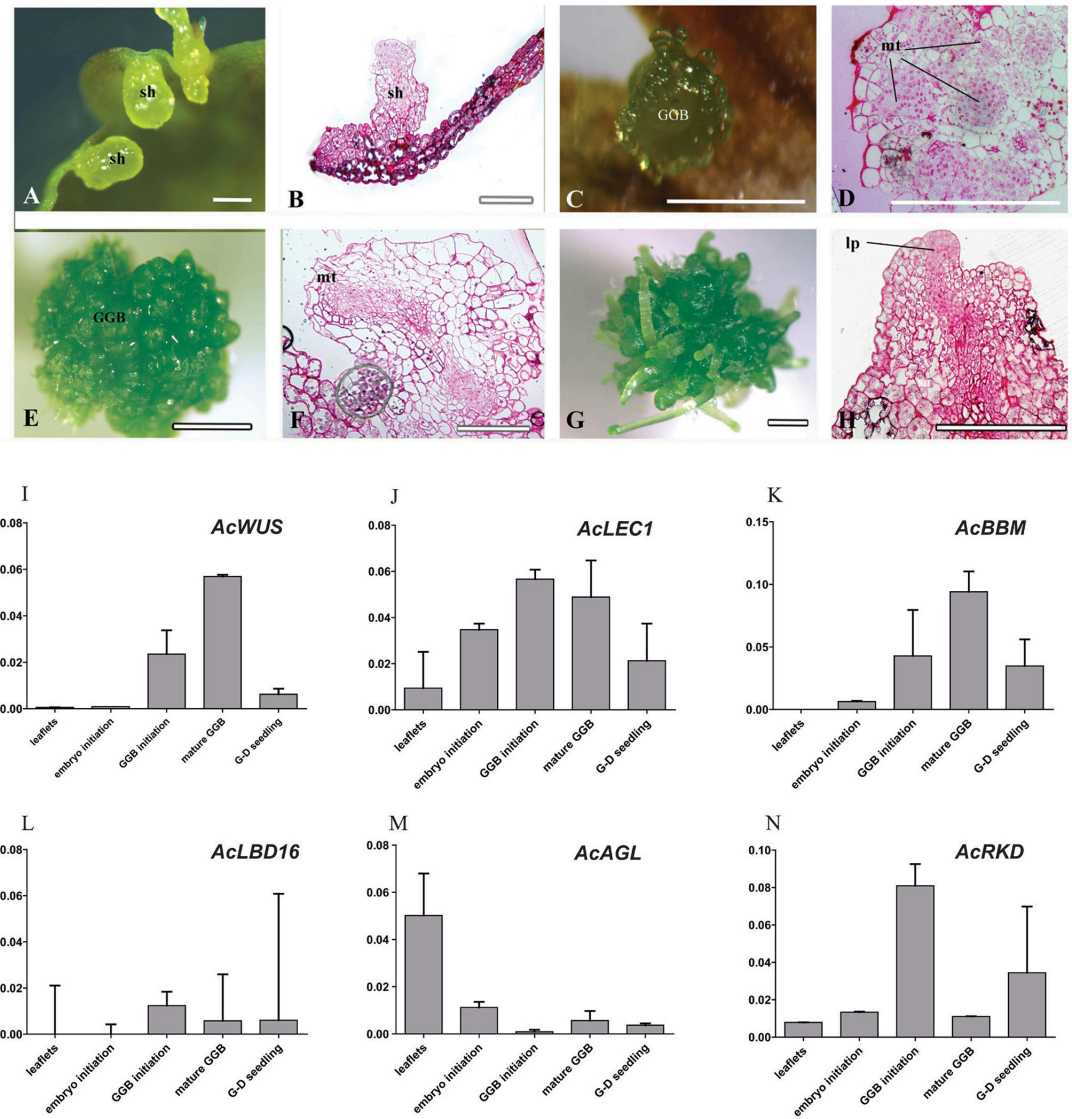
**Cyto-morphological and gene expression of adventitious shoots and GGBs**. **(A)** Shoots formed on medium without PGR. **(B)** Histological section of initiated shoots (14-day). **(C)** Initiated GGBs (14-day). **(D)** Vertical section of initiated GGBs. **(E)** Mature GGBs (40-day). **(F)** Vertical section of mature GGBs (40-day). **(G)** GGB-derived shoots cultured on medium without PGR. **(H)** Vertical section of GGB-derived shoots. **(I–N)** Expression of *AcWUS, AcLEC1, AcBBM, AcLBD16, AcAGL, AcRKD* during shoots and GGBs-derived somatic embryogenesis. dcz, dividing cells zone; GGB, green globular body; lp, leaf primordium; mt, meristematic tissue. Bars = 500 μm.

Adventitious shoots compacted with some trichomes had obvious shoot apices, leaves and roots (Figures 1E,2A,B). The globular dark-green GGBs appeared 2 weeks after the incubation of explants (Figures 1B,F,2C). Multiple meristematic zones were found inside the initial GGB tissue (Figure 2D). Thereafter, many hair-like adventitious shoots formed on the surface of GGBswhen they were moved to the 2, 4-D-free medium with lower BA (Figures 2E–H). By the means of qRT-PCR analysis, we examined the expression patterns of six selected genes during *A. capillus-veneris* direct SE. As our results showed, *AcLEC1* is functionally pleiotropic, and it was highly expressed in the initiation phase of embryos and GGBs, and mature GGBs, respectively (Figure 2J). However, the expression decreased in GGB-derived seedlings as the shoots continued to regenerate (Figure 2J). *AcWUS* and *AcBBM* had similar expression patterns with expression peak in the mature GGBs and low point in embyro initiation (Figures 2I,K). *AcRKD* was only highly expressed at GGB initiation stage during the direct SE (Figure 2N). Comparing to the explant leaflets, expressions of *AcLBD16* and *AcAGL* were rarely detectable in shoot and GGB development phases (Figures 2L,M).

### Callus induction and callus-derived shoot organogenesis

**Figure 3:**
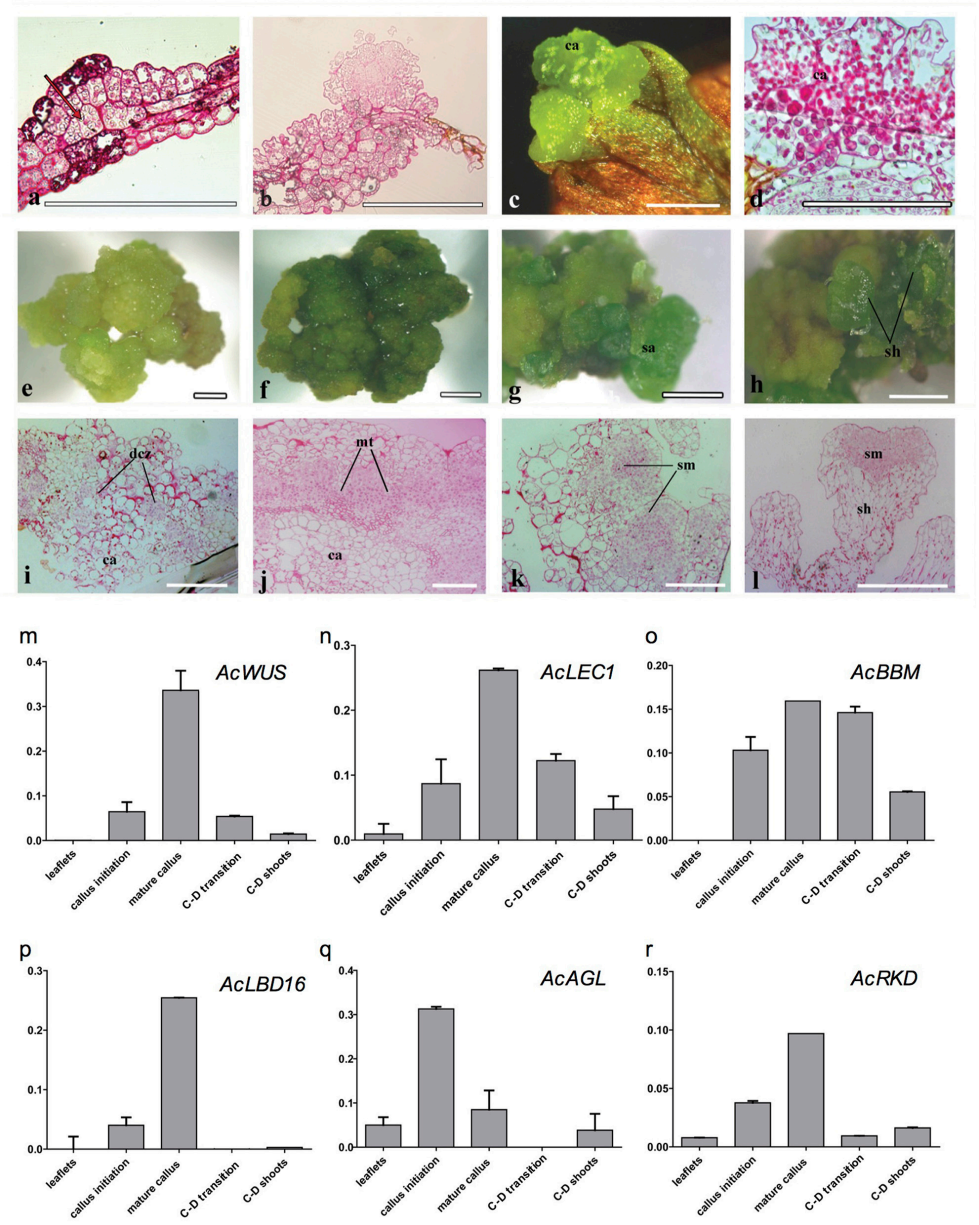
**Cyto-morphological and gene expression during callus-derived somatic embryogenesis. (a)** The callus originated from the mesophyll cells. **(b)** Primary callus lump of the leaf. **(c)** Initiated callus (14-day) formed on medium with 0.5 mg/L 2,4-D and 0.5 mg/L 6-BA. **(d)** Vertical section of initiated callus. **(e)** Mature callus (40-day) on medium with 0.5 mg/L 2,4-D and 0.5 mg/L 6-BA. **(i)** Vertical section of mature callus. **(f**–**h)** Callus-embryo transition on medium with 0.5 mg/L BA. **(j**–**k)** Vertical section of callus showing meristemoids. **(l)** Vertical section showing shoot meristem. **(m**–**r)** Expression of *AcWUS, AcLEC1, AcBBM, AcLBD16, AcAGL, AcRKD* during callus-derived somatic embryogenesis. dcz, dividing cells zone; ca, callus; mt, meristematic tissue; sa, shoot apical; sh, shoot; sm, shoot meritem. Bars = 500 μm.

Histological analysis revealed that the callus originated from the mesophyll cells, especially those closely connected to marginal veins of the pinnately lobes of explant leaflets (Figure 3a). The callus, an undifferentiated mass of friable light green tissue, lacked embryo-like structures or apical meristems (Figures 3b–d). The best production (>90%) of calli was found on media with 0.5 mg/L 2,4-D and 0.5–1.0 mg/L BA, where there was no significant difference in the callus productivity between hormone combinations in the two basal media (Table S1). When growing to ~1 cm in diameter, calli were cut into small pieces (~3 mm in diameter) and then transplanted into the same medium for subculture. Their cells divided actively and gave rise to fresh light green calli with multiple meristemoid zones after ~1 month inoculation (Figures 3e,i). Table S1 showed the effect of BA on shoot regeneration from calli. Among various PGRs tested, the highest frequency of shoot regeneration from calli (60–70%) and number of shoots per callus (4–5) were observed on either MS or ½ MS medium containing 0.5 mg/L BA (Table 1). After the first 40 days of culture, calli became compact and green (Figure 3f). At this time, meristemoids were produced inside rather than on the surface of the calli and shoot primordia formed (Figure 3j). After 3 months' culture, each callus produced several shoots (Figures 3g–l). During this indirect SE, expressions of *AcWUS, AcLEC1, AcLBD16* and *AcRKD* were clearly detected in callus development phases, with the maximum expression in mature calli (40-day calli), and then decreased (Figures 3m,n,p,r). *AcAGL* expressed during callus formation with higher expression levels at callus initiation (14-day calli) and in mature calli (40-day calli) (Figure 3q). Up-regulated expression of *AcBBM* was detected in the entire callus development and reached apeakat mature callus stage (Figure 3o). It was also highly expressed during the callus-embryo transition (C-D transition), but showed a low expression when shoots became visible (C-D shoots) (Figure 3o).

**Table 1:** **Callus-based shoot regeneration of *A. capillus-veneris***.

**Treatment BA (mg/L)**	**Percentage of callus producing shoots (%)^1^**		**Average number of buds per callus^1^**	
	**MS**	**1/2 MS**	**MS**	**1/2 MS**
0	23.33^abc^ ± 8.89	26.67^abc^ ± 4.44	0.63^A^ ± 0.11	0.70^A^ ± 0.13
0.5	63.33^e^ ± 8.89	76.67^f^ ± 4.44	4.20^E^ ± 0.38	5.00^F^ ± 0.20
1.0	36.67^cd^ ± 4.44	46.67^d^ ± 4.44	3.33^D^ ± 0.22	4.10^E^ ± 0.13
1.5	30.00^bc^ ± 0	33.33^bcd^ ± 0.04	1.93^C^ ± 0.22	2.07^C^ ± 0.04
2.0	13.33^a^ ± 4.44	20.00^ab^ ± 6.67	0.30^A^ ± 0.07	1.27^B^ ± 0.11

*Data show mean ± SE of three replicates, each comprising 9–10 explants. Different letters in a column indicate significant differences at P < 0.05 according to Duncan's multiple range test*.

1 *The diameter of the calli was 8–10 mm*.

### Identification and phylogenetic analysis of *AcWUS, AcBBM, AcLBD16, AcAGL, and AcRKD*

**Figure 4:**
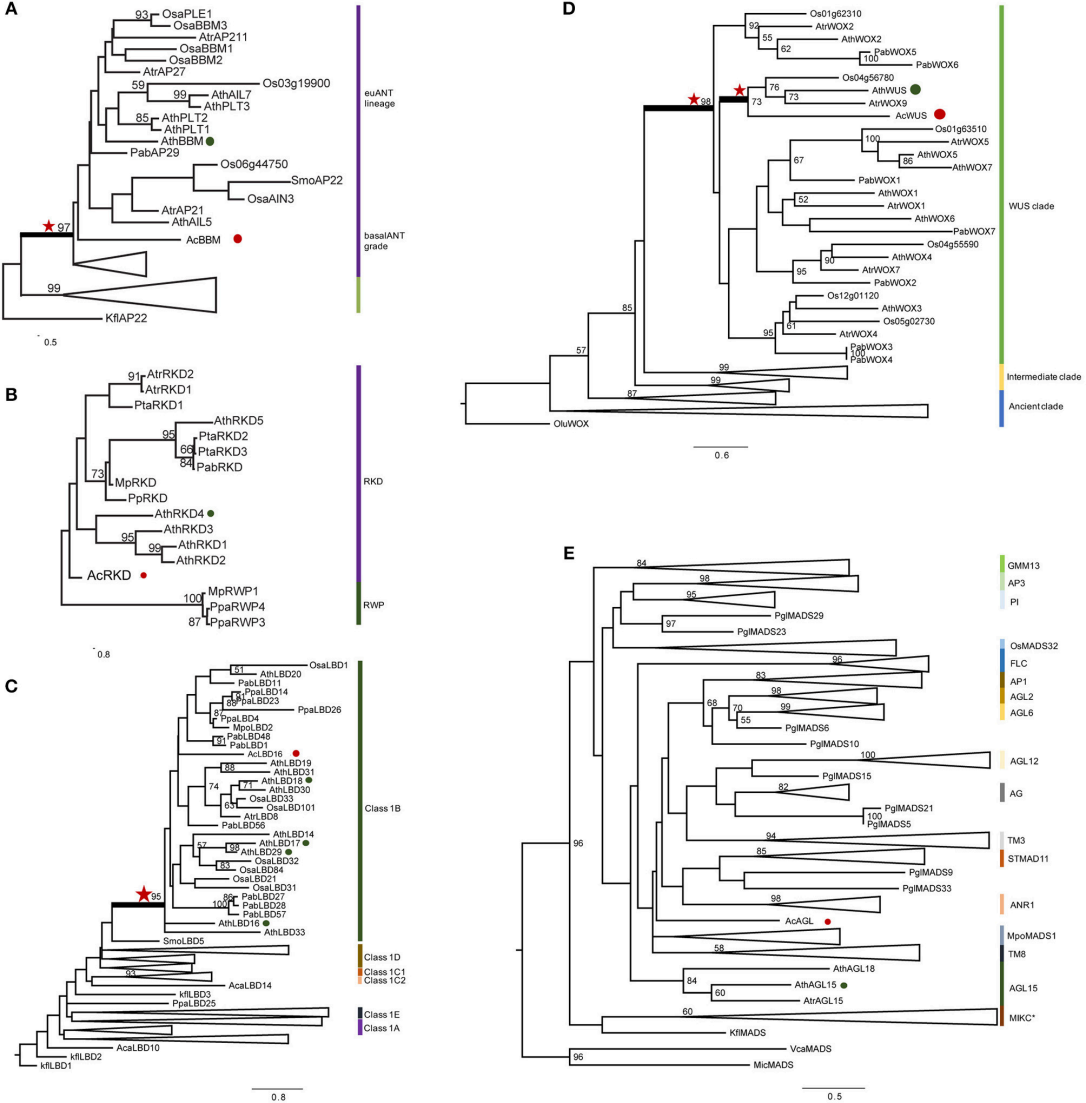
**Phylogenetic analysis of *AcWUS, AcBBM, AcLBD16, AcAGL, and AcRKD***. Maximum-likelihood tree of five gene family proteins constructed using sequences of *A. capillu-veneris* in this study and other sequences of representative plant species. See Table S4 for sequences and accession numbers. Numbers at the branches indicate bootstrap values calculated from 1,000 replicates. Only values higher than 50 are shown. In each tree, the different clades are indicated by colored boxes and named. Red stars refer homologous genes with high confidence. Red circles refer the genes from *A. capillus-veneris*. Green circles refer the genes from *A. thaliana*. **(A)** AcBBM, **(B)** AcRKD, **(C)** AcLBD16, **(D)** AcWUS, and **(E)** AcAGL.

Phylogenetic analyses were performed to analyze evolution of *AcWUS, AcBBM, AcLBD16, AcAGL*, and *AcRKD* and their homologs in other green plants (Figure 4). The gene trees showed that these genes and their homologs of other green plants formed monophyletic clades, respectively (Figure 4). However, tree topologies were also found for these gene families, suggesting that they could experience different evolutionary trajectories during plant evolution.

The phylogenetic tree of *WOX* gene family showed that there were three clades, and the *AcWUS* was grouped with the *WUS* counterparts as a strong supported clade (Figure 4D). Within the *WUS* clade, *AcWUS* showed high sequence similarity to Arabidopsis *AtWUS*. The *AcBBM* was identified as a member of the *APETALA2 (AP2)/ETHYLEN-RESPONSE-FACTOR (ERF)* gene family. The phylogenetic tree of *BBM* genes showed that pteridophyte *BBMs*, including *AcBBMs*, were grouped at the base of the seed plant euANT lineage (Figure 4A). Also, phylogenetic analysis of *AcLBD* and its counterparts of other plants indicated that they all possess the signature motif of Class IB. In addition, phylogenetic tree showed that *AcLBD16* gene, though scattering among seed plant clades, resided in the Class 1B clade with strong support (Figure 4C). Hence, *AcLBD16* may be a putative ortholog of Arabidopsis *LBD16, LBD17, LBD18, LBD29*. A maximum-likelihood tree constructed from the whole RKD protein sequences showed that all the plant RKDs were clustered in a single clade which was well separated from the outgroup RWP-RK proteins (Figure 4B). *AcRKD* had high sequence similarity with Arabidopsis *RKDs* at the RWP-RK and carboxy-terminal domains. In addition, the phylogenetic position of *AcAGL* remains uncertain based on our analysis on the MADS Type II sequences using RAxML (Figure 4E). Seventeen branches (MIKC^*^, AGL2/AGL6/FLC/SQUA, DEF/GLO/OsMADS32/GGM13, AGAMOUS, AGL12, AGL15, AGL17, GpMADS4, StMADS11, TM3, and TM8) can be defined in the tree, but *AcAGL* had a close relationship with *MpoMADS1* and *TM8* rather than with *AGL15* of Arabidopsis and *Amborella trichopoda* (Figure 4E).

## Discussion

### Shoot regeneration *via* somatic embryogenesis

An efficient *in vitro* regeneration system *via* SE was developed for *A. capillus-veneris*. Direct SE via adventitious shoots and green globular bodies (GGBs), and indirect SE *via* calli were obtained with the maximal rate on the MS media. Shoot regeneration *via* direct SE is usually initiated by plant growth regulators (PGRs) although their usage is rare (Fernández et al., 1997; Bertrand et al., 1999). *In vitro* adventitious shoot initiation was found on the medium with very low PGR concentration or without any PGRs (Beck and Caponetti, 1983; Table S1). GGBs became dominant with the increase of BA concentration. Amaki and Higuchi (1991) reported that the optimal medium for GGB proliferation of *Adiantum* was ½ MS with 1.0 mg/L BA, whereas our data showed that MS or ½ MS medium with a higher concentration of BA (2.0 mg/L) obtained the best proliferation.

Indirect SE, especially callus induction, has rarely been successful in ferns. In the present study, we found that the medium containing 0.5 mg/L 2,4-D and 0.5–1.0 mg/L BA is the most efficient one for the callus induction of *A. capillus-veneris* (Table S1). This result is in agreement with Byrne and Caponetti (1992), who reported that 2,4-D and sucrose were necessary to produce calli in the Boston fern, *Nephrolepis exaltata*. Calli are very sensitive to *in vitro* culture conditions and easily turn brown (Ahloowalia, 1982; Northmore et al., 2012). It has been reported to be difficult for generating intact sporophytes from a callus in ferns (Kwa et al., 1997; Byrne and Caponetti, 1992). This is in line with our results on the frequency of shoot organogenesis of *A. capillus-veneris* (Table 1). The morphogenetic pathway could be important for improving the regeneration rate (Fernández and Revilla, 2003). The meristemoid tissue found in the indirect SE of *A. capillus-veneris* is critical for organogenesis of a callus *in vitro*, which has not previously been described in ferns (Attfield and Evans, 1991; Bobák et al., 1993; Ovečka et al., 2000).

### Phylogenetic relationships of embryogenesis-associated genes in land plants

Although only six embryogenesis-associated genes were studied in the present study, phylogenetic analyses of *AcBBM, AcWUS, AcAGL, AcLBD16*, and *AcRKD* of *A. capillus-veneris* showed that all those gene homologs can be found in other land plants, hardly in algae. The homologous genes were generally clustered as a clade in their respective phylogenetic trees (Figure 4), suggesting that the embryogenesis-associated genes could originate and evolve with colonization of plants to terrestrial environments. However, the mechanisms behind these evolutionary events remain unresolved. WUSCHEL-related homeobox (WOX) members contain a conserved homeodomain essential for plant development by regulating cell division and differentiation (Zuo et al., 2002; Li et al., 2013). So far, there has been no data concerning evolution and function of fern WUS homologs. Our phylogenetic analysis revealed that AcWUS is a putative ortholog of Arabidopsis WUS with high bootstrap value. In addition, the WUS clade was the latest derived lineage in the phylogenetic tree of WOX family genes (Figure 4D), which is consistent with previous findings in Arabidopsis as well as other plants (Lian et al., 2014). The RKD family of plant RWP-RK factors is expressed in reproductive cells of land plants, and has a single origin (Koi et al., 2016). In the gene trees (Figures 4B,C), AcRKD resided in the RKD clade corresponding to the RKD subfamily designated by Chardin et al. (2014); AcLBD is located in the Class IB LBD gene lineage, which was reported to be involved in root development (Coudert et al., 2012; Chanderbali et al., 2015). The BBM genes were described in *A. capillus-veneris* for first time. Our analysis showed that AcBBM shares high similarity with BBMs in other land plants, and is imbedded in the clade euANT (Figure 4A). All of these suggested that the embryogenesis-associated genes were highly conserved, and they originated early, at least earlier than occurrence of ferns.

### Expression of six embryogenesis-associated genes during shoot regeneration

**Figure 5:**
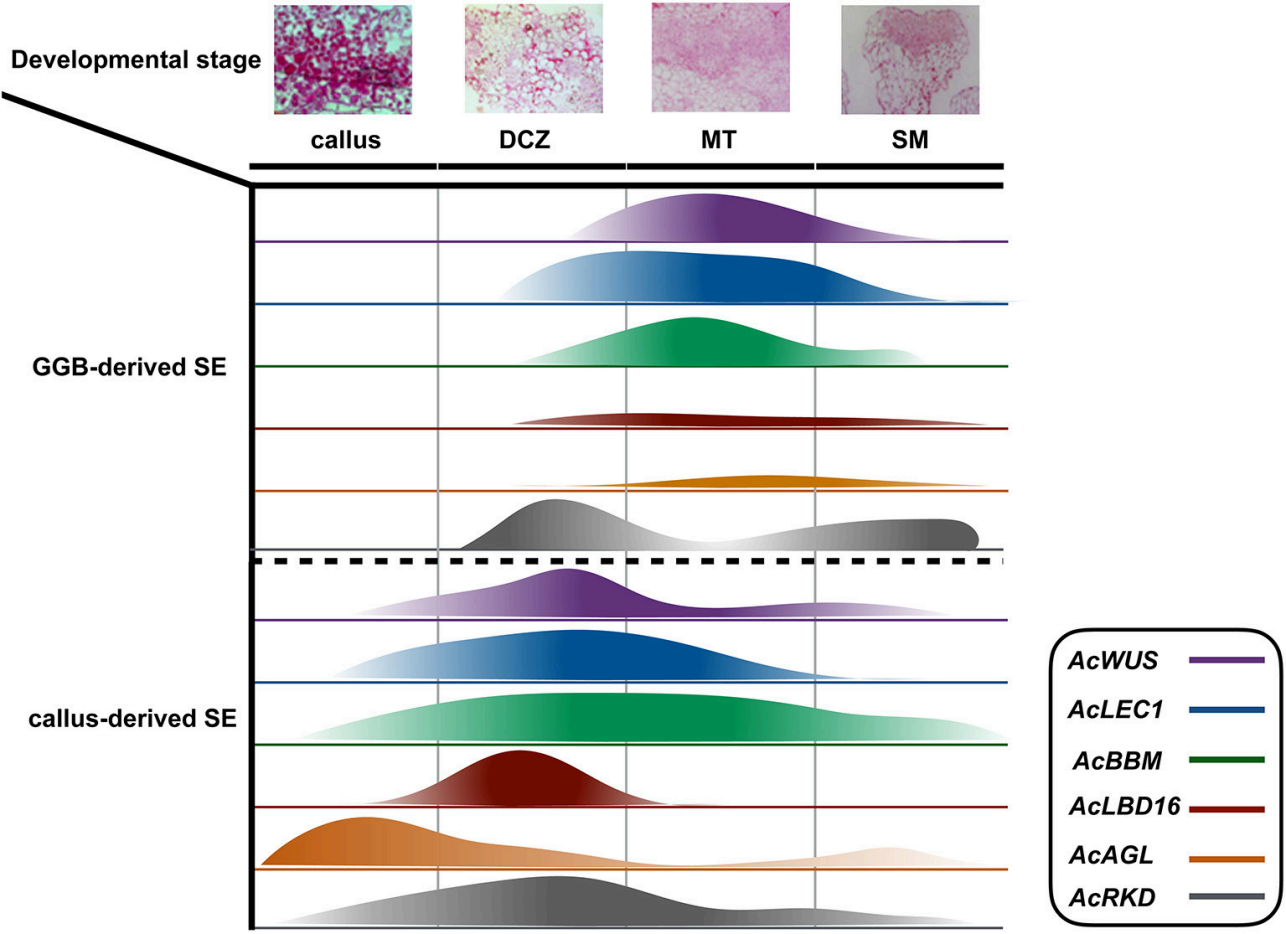
**Schematic diagram of the expression of *AcWUS, AcLEC1, AcBBM, AcLBD16*, *AcAGL*, and *AcRKD* during somatic embryogenesis in *Adiantum capillu-veneris***. Four important developmental stages callus, dividing cells zone (dcz), meristematic tissue (MT), shoot meritem (SM) were chosen and the width of the bars represents the results of those genes' expression.

Embryogenesis-associated genes have been extensively characterized in carrot and Arabidopsis by using SE (Ikeuchi et al., 2013; Radoeva and Weijers, 2014), but less have been evaluated in ferns. In this study, the putative orthologs of Arabidopsis *BBM, LEC1, WUS, AGL15, LBD16*, and *RKD4* were identified and cloned from *A. capillus-veneris*. The developmental stage-specific expression patterns of all these six genes during GGBs and calli-derived SE are shown in Figure 5. We found the expression pattern of each gene is in agreement with that of its counterparts in angiosperms. For instance, expression of *AcLBD16* in callus development is consistent with the expression patterns of Arabidopsis *LBD16–18* and *LBD29*, and poplar *PtaLBD1*, which are sufficient to promote callus formation under auxin conditions (Yordanov et al., 2010; Fan et al., 2012). Our analyses showed that *AcBBM* was expressed during PGR-induced embryogenesis or callus formation (Figure 5), similar to the reports on orthologs of Arabidopsis, *Brassica napus* and *Glycine max* (Boutilier et al., 2002; El Ouakfaoui et al., 2010). It is well documented in many species that *WUS* and *WOX* genes regulate the shoot meristem cells, and their overexpression can induce callus formation (Mayer et al., 1998; Loyola-Vargas and Ochoa-Alejo, 2016). During the SE of *A. capillus-veneris, AcWUS* was detected with high expression levels in GGB and callus developments. This finding, together with the observation of somatic embryos in Arabidopsis as well as *WUS* expression in several callus lines suggests that the *WUS* genes may be involved in the regulation of both meristematic and embryogenic cells (Zuo et al., 2002; Iwase et al., 2011; Fehér, 2015). *AcRKD* was highly expressed in GGB induction and callus development (Figure 5), a phenomenon consistent with the result of ectopic overexpression of *RKD1* and *RKD2* that induced callus development without PGRs, and the expression of *RKD4* in early embryos (Waki et al., 2011).

### Key regulatory genes involved in embryogenesis in land plants

**Figure 6:**
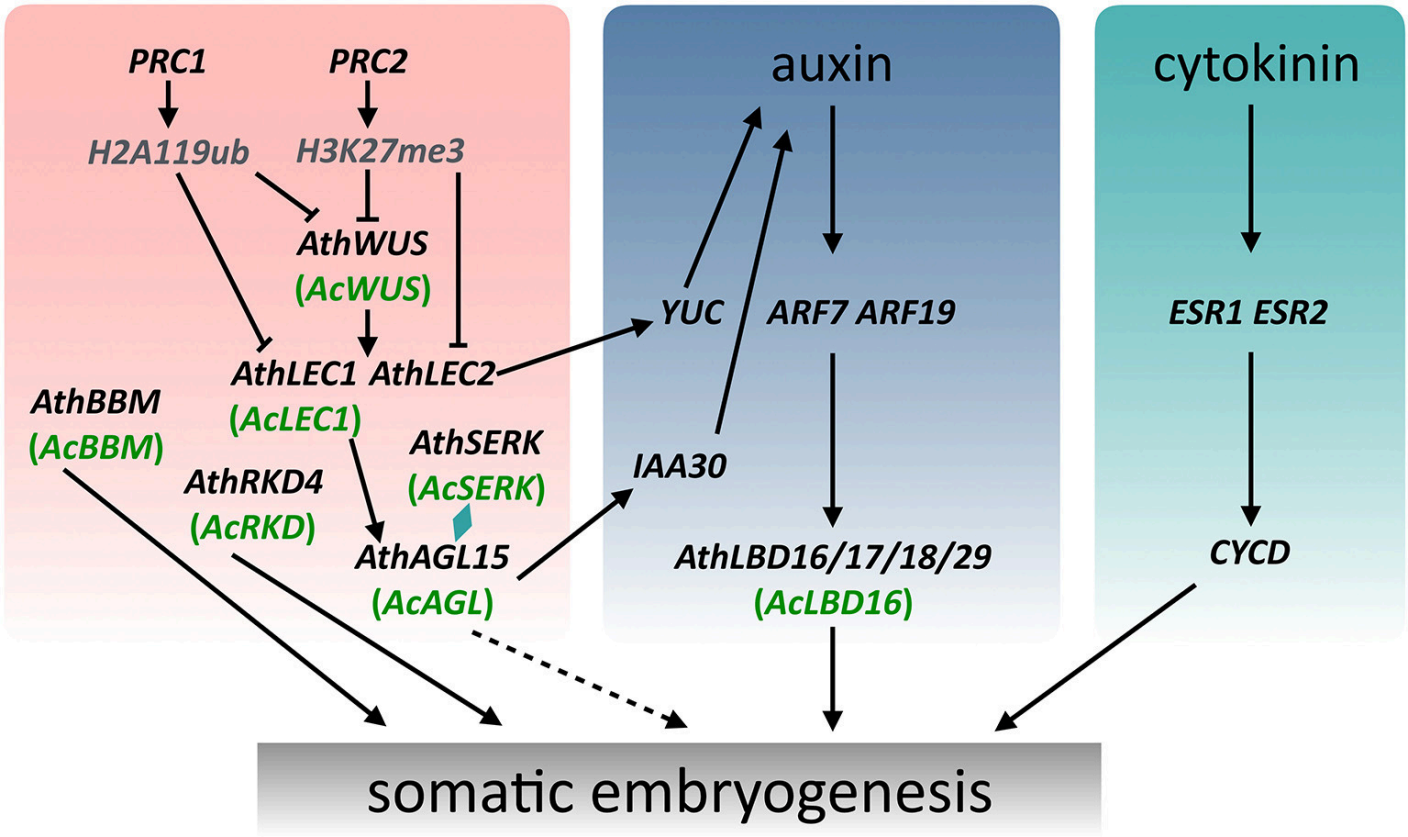
**A schematic model showing the regulation of somatic embryogenesis in Arabidopsis and *Adiantum capillus-veneris***. The WUS expression subsequently induces expression of LEC1 and LEC2, which together with AGL15 and SERK modulate the endogenous levels of auxin to promote somatic embryogenesis. Arrows with a solid line indicate direct transcriptional regulation by molecular evidence. Arrows with a dotted line indicate transcriptional regulation that mechanisms are not clear.

SE, in response to exogenous and/or endogenous signals, has been studied and applied in plants for more than 50 years (Radoeva and Weijers, 2014; Fehér, 2015; Loyola-Vargas and Ochoa-Alejo, 2016), but the molecular mechanisms initiating and controlling this process remain unclear. As the ultimate product of the SE is similar, it can be expected that the basic regulatory mechanism involved in this process is very conserved during plant evolution. Numerous molecular studies have identified many regulatory genes and gain the entry into the regulatory networks underling the SE processes of various plant species. Based on previous and present studies (Radoeva and Weijers, 2014; Fehér, 2015; Ikeuchi et al., 2015; Guan et al., 2016; Loyola-Vargas and Ochoa-Alejo, 2016), we summarized a basic graphic illustration of the regulatory networks controlling SE (Figure 5). Although promising progress in characterizing the molecular mechanisms of SE has been made in seed plants, but little is known about the regulatory genes and mechanisms of this process in ferns. Compared with some model plants such as Arabidopsis and carrot, it is more difficult to understand the molecular mechanism of the fern SE due to a lack of effective defective mutants, genome sequence data, and different phylogenetic positions in the evolution of land plants. Even so, the identification and expression analysis of key SE-associated genes in *A. capillus-veneris* can provide a global view of transcriptional events important for GGB-/callus-derived SE in this species, and will help us to understand the regulatory networks of SE, even of ZE in ferns. These data, coupled with the phylogenetic analyses of six regulatory genes, offer new information for a better understanding of the fern SE and the evolution of key regulatory genes associated with embryogenesis in land plants (Figure 6).

### Evolution of reproductive organs: embryos to seeds

Reproduction in land plants can be viewed as a complex, partly hierarchical, series of developmental processes, which together with their underlying genetic regulators produce morphological innovations, such as embryos, seeds and flowers. Adaptation of land plants to terrestrial environments occurs as the variation in genetic and developmental processes is winnowed by selection (Crane and Kenrick, 1997; Pires and Dolan, 2012). Embryo formation is the first innovation acquired by land plants during evolution, and followed by the development of seeds (Becker and Marin, 2009; Radoeva and Weijers, 2014). These two developmental processes are closely connected, and the former is a prerequisite for the latter. Although the two processes could be controlled by different developmental pathways, they probably share many regulatory genes. For example, in Arabidopsis, *LEC1* is not only involved in the embryogenesis, but also regulates seed maturation (Goldberg et al., 1994; Harada, 1997; Radoeva and Weijers, 2014); the up-regulated expression of *AcLEC1*, a *LEC1* homolog in *A. capillus-veneris*, can facilitate the formation of seed-like traits, such as the accumulation of nutrient reserves and delayed development of embryos in this fern species under some laboratory conditions (Fang et al., unpublished data), which are completely similar to the seed traits produced in Arabidopsis. All the those suggests that the embryogenesis-associated genes could be co-optioned to a new developmental program, which produces morphological innovations, like formation of seed-like traits in *A. capillus-veneris*. Thus, seed formation may have resulted from a newly built regulatory network, which is established by cooption and modification of existing genes or networks.

## Author contributions

GR, XL, and SB designed the experiments. XL and JH performed the experiments. XL, JH, and YF analyzed the data. XL and GR wrote the manuscript. All authors read and approved the final manuscript.

### Conflict of interest statement

The authors declare that the research was conducted in the absence of any commercial or financial relationships that could be construed as a potential conflict of interest.

## Supplementary material

Table S1 **Effect of different media on induction rates of shoots, GGBs, and calli**.

Table S2 **Primers used for the sequences verified**.

Table S3 **Primers used for the qRT-PCR reaction**.

Table S4 **MADS-box family sequences and accession numbers**.
