# Supplementary material for: Expression Analyses of Embryogenesis-Associated Genes during Somatic Embryogenesis of Adiantum capillus-veneris L. In vitro: New Insights into the Evolution of Reproductive Organs in Land Plants
Source: Front Plant Sci. 2017 Apr 27;8:658. doi: 10.3389/fpls.2017.00658 (PMC5406782; doi:10.3389/fpls.2017.00658)
Supplement: Table S1 — Effect of different media on induction rates of shoots, GGBs, and calli. [file Table1.DOCX]

| **Treatment**  **BA+2,4-D (mg/L)** | **% induction** | | | | | |
| --- | --- | --- | --- | --- | --- | --- |
|  | MS | | | ½ MS | | |
|  | **Shoot** | **GGB** | **Callus** | **Shoot** | **GGB** | **Callus** |
| 0 + 0 | **87.50^a^±0** | 0^a^ | 0^a^ | **79.45^a^±8.05** | 0^a^ | 0^a^ |
| 0.5 + 0 | **90.9^a^±9.09** | 14.55^b^±5.46 | 0^a^ | **83.77^a^±1.95** | 23.38^b^±5.19 | 0^a^ |
| 1.0 + 0 | **87.50^a^±12.50** | 38.75^cd^±1.25 | 0^a^ | **66.97^a^±4.47** | 47.30^c^±9.80 | 0^a^ |
| 1.5+ 0 | 33.04^b^±4.46 | **73.22^e^±1.79** | 0^a^ | 37.50^b^±0 | **75.00^d^±0** | 6.25^a^±6.25 |
| 2.0+0 | 0^c^ | **100^f^±0** | 0^a^ | 0^c^ | **100^e^±0** | 7.15^ab^±7.15 |
| 0 + 0.5 | 0^c^ | 0^a^ | **85.42^h^±2.09** | 0^c^ | 0^a^ | **82.86^g^±2.86** |
| 0.5 + 0.5 | 0^c^ | 0^a^ | **93.75^h^±6.25** | 0^c^ | 0^a^ | **96.16^g^±3.85** |
| 1.0 + 0.5 | 0^c^ | 0^a^ | **96.16^h^±3.85** | 0^c^ | 0^a^ | **95.00^g^±5.00** |
| 1.5+0.5 | 0^c^ | 34.85^c^±1.50 | 40.40^def^±4.04 | 0^c^ | 41.67^bc^±8.34 | 40.97^def^±3.47 |
| 2.0+0.5 | 0^c^ | 48.57^d^±8.57 | 36.43^def^±6.43 | 0^c^ | 51.25^c^±11.25 | 43.75^ef^±6.25 |
| 0 + 1.0 | 0^c^ | 0^a^ | 44.51^ef^±1.65 | 0^c^ | 0^a^ | 50.00^ef^±7.14 |
| 0.5 + 1.0 | 0^c^ | 0^a^ | 50.86^fg^±4.71 | 0^c^ | 0^a^ | 50.00^ef^±7.14 |
| 1.0 + 1.0 | 0^c^ | 0^a^ | 66.67^g^±0 | 0^c^ | 0^a^ | 61.25^f^±11.25 |
| 1.5+ 1.0 | 0^c^ | 0^a^ | 25.00^bcd^±5.00 | 0^c^ | 0^a^ | 38.89^def^±5.56 |
| 2.0+ 1.0 | 0^c^ | 10.56^ab^±0.56 | 31.67^cdef^±1.67 | 0^c^ | 0^a^ | 51.58^ef^±3.98 |
| 0+ 1.5 | 0^c^ | 0^a^ | 7.15^ab^±7.15 | 0^c^ | 0^a^ | 43.65^ef^±0.79 |
| 0.5+ 1.5 | 0^c^ | 0^a^ | 35.42^def^±2.09 | 0^c^ | 0^a^ | 33.75^cde^±0.79 |
| 1.0+ 1.5 | 0^c^ | 0^a^ | 33.64^def^±6.39 | 0^c^ | 0^a^ | 43.65^ef^±6.35 |
| 1.5+ 1.5 | 0^c^ | 0^a^ | 47.22^ef^±2.78 | 0^c^ | 0^a^ | 41.67^ef^±8.34 |
| 2.0+ 1.5 | 0^c^ | 0^a^ | 36.43^def^±6.43 | 0^c^ | 0^a^ | 42.78^ef^±12.78 |
| 0+ 2.0 | 0^c^ | 0^a^ | 0^a^ | 0^c^ | 0^a^ | 11.81^abc^±0.70 |
| 0.5+ 2.0 | 0^c^ | 0^a^ | 5.00^a^±5.00 | 0^c^ | 0^a^ | 18.75^abc^±6.25 |
| 1.0+ 2.0 | 0^c^ | 0^a^ | 29.77^cde^±13.10 | 0^c^ | 0^a^ | 18.75^abcd^±6.25 |
| 1.5+ 2.0 | 0^c^ | 0^a^ | 11.11^ab^±11.11 | 0^c^ | 0^a^ | 29.29^cde^±0.72 |
| 2.0+ 2.0 | 0^c^ | 0^a^ | 14.59^abc^±2.09 | 0^c^ | 0^a^ | 28.47^bcde^±15.97 |
| Means marked by the same letter within a column were not significantly different at *P* < 0.05  Data shown mean ± SE of two replicates, each comprising 8–11 explants | | | | | | |

**Table 1. Effect of different media on induction rates of shoots, GGBs and calli.**
