# Supplementary material for: Expression Analyses of Embryogenesis-Associated Genes during Somatic Embryogenesis of Adiantum capillus-veneris L. In vitro: New Insights into the Evolution of Reproductive Organs in Land Plants
Source: Front Plant Sci. 2017 Apr 27;8:658. doi: 10.3389/fpls.2017.00658 (PMC5406782; doi:10.3389/fpls.2017.00658)
Supplement: Table S2 — Primers used for the sequences verified. [file Table2.DOC]

**Table 2 Primers used for the sequences verified.**

| Full-length cDNA clone | | |
| --- | --- | --- |
| *AcLEC1* | Primer-S | 5’-ATGGCTTATCGCCATTCCAGTCC-3’ |
|  | Primer-A | 5’- TCAGGCTCCTCTCTGCTGCCCA-3’ |
| *AcWUS* | Primer-S | 5’-ATGGCAGAGCAATGCTGTCGAG-3’ |
|  | Primer-A | 5’-TCAGTGATCAACGGCAAGCTT-3’ |
| *AcLBD16* | Primer-S | 5’-ATGAATCAAGAGGGGGAGCAAG-3’ |
|  | Primer-A | 5’- TCAAGTAGAGTTATTGAGTTT-3’ |
| *AcAGL* | Primer-S | 5’-ATGGCGCTGCCTGCCTCCGAC-3’ |
|  | Primer-A | 5’-TCATCGACTGAAGGCGCAGGC-3’ |
| *AcBBM* | Primer-S | 5’-TTCTCACTGATGAACTCCGCA-3’ |
|  | Primer-A | 5’-CTACTGATCCCATGAAGCAAA-3’ |
| *AcRKD* | Primer-S | 5’-ATGGGCGCCCATCTCCATAGCC-3’ |
|  | Primer-A | 5’-CTAAAGGAAATGATCCGTTGT-3’ |
