# Supplementary material for: Expression Analyses of Embryogenesis-Associated Genes during Somatic Embryogenesis of Adiantum capillus-veneris L. In vitro: New Insights into the Evolution of Reproductive Organs in Land Plants
Source: Front Plant Sci. 2017 Apr 27;8:658. doi: 10.3389/fpls.2017.00658 (PMC5406782; doi:10.3389/fpls.2017.00658)
Supplement: Table S3 — Primers used for the qRT-PCR reaction. [file Table3.DOCX]

**Table 3 Primers used for the qRT-PCR reaction.**

| qRT-PCR reaction | | |
| --- | --- | --- |
| *AcLEC1* | Primer-qS | 5’- GTCTCCAAGCCGAGCTAAAT -3’ |
|  | Primer-qA | 5’- GTCTCCAAGCCGAGCTAAAT -3’ |
| *AcWUS* | Primer-qS | 5’- GAGCGATGGTGCGGATATT -3’ |
|  | Primer-qA | 5’- AGAGCTGGAGACCCTGTATT -3’ |
| *AcLBD16* | Primer-qS | 5’- GTCTCCAAGCCGAGCTAAAT -3’ |
|  | Primer-qA | 5’- ATCTGATGGCCAAGGAACTG -3’ |
| *AcAGL* | Primer-qS | 5’- ACAAAGTCCGTGCGGTAAA -3’ |
|  | Primer-qA | 5’- TGAGGACTCTTGGACCTCTT -3’ |
| *AcBBM* | Primer-qS | 5’-CAGCAGAACCCATCCTCAAA -3’ |
|  | Primer-qA | 5’- AACGAAGGTGCCCATCATAC -3’ |
| *AcRKD* | Primer-qS | 5’- TGCTGAATGTGGTGAGAGAG -3’ |
|  | Primer-qA | 5’- GAGCCCTACAGGAGCATAAAT -3’ |
| *AcACTIN* | Primer-qS | 5’-ACCACTGCAGAAAGGGAAATTG-3’ |
|  | Primer-qA | 5’-CTGGAAAAGGACCTCAGGACAT-3’ |
